# Molecular reassessment of diaporthalean fungi associated with strawberry, including the leaf blight fungus, *Paraphomopsis obscurans* gen. et comb. nov. (*Melanconiellaceae*)

**DOI:** 10.1186/s43008-021-00069-9

**Published:** 2021-06-22

**Authors:** Dhanushka Udayanga, Shaneya D. Miriyagalla, Dimuthu S. Manamgoda, Kim S. Lewers, Alain Gardiennet, Lisa A. Castlebury

**Affiliations:** 1 Department of Biosystems Technology, Faculty of Technology, University of Sri Jayewardenepura, Pitipana, Homagama, 10200 Sri Lanka; 2 Department of Botany, Faculty of Applied Sciences, University of Sri Jayewardenepura, Nugegoda, 10250 Sri Lanka; 3 Genetic Improvement of Fruits and Vegetables Laboratory, United States Department of Agriculture Agricultural Research Service, Beltsville, MD 20705 USA; 4 Société Mycologique Issoise, 14 rue Roulette, F-21260 Véronnes, France; 5 Mycology and Nematology Genetic Diversity and Biology Laboratory, United States Department of Agriculture Agricultural Research Service, Beltsville, MD 20705 USA

**Keywords:** foliar fungi, *Fragaria*, leaf blotch, plant pathogens, petiole blight, Sordariomycetes, one new taxon

## Abstract

Phytopathogenic fungi in the order *Diaporthales* (*Sordariomycetes*) cause diseases on numerous economically important crops worldwide. In this study, we reassessed the diaporthalean species associated with prominent diseases of strawberry, namely leaf blight, leaf blotch, root rot and petiole blight, based on molecular data and morphological characters using fresh and herbarium collections. Combined analyses of four nuclear loci, 28S ribosomal DNA/large subunit rDNA (*LSU*), ribosomal internal transcribed spacers 1 and 2 with 5.8S ribosomal DNA (*ITS*), partial sequences of second largest subunit of RNA polymerase II (*RPB2*) and translation elongation factor 1-α (*TEF1*), were used to reconstruct a phylogeny for these pathogens. Results confirmed that the leaf blight pathogen formerly known as *Phomopsis obscurans* belongs in the family *Melanconiellaceae* and not with *Diaporthe* (syn. *Phomopsis*) or any other known genus in the order. A new genus *Paraphomopsis* is introduced herein with a new combination, *Paraphomopsis obscurans*, to accommodate the leaf blight fungus. *Gnomoniopsis fragariae* comb. nov. (*Gnomoniaceae*), is introduced to accommodate *Gnomoniopsis fructicola*, the cause of leaf blotch of strawberry. Both of the fungi causing leaf blight and leaf blotch were epitypified. Fresh collections and new molecular data were incorporated for *Paragnomonia fragariae* (*Sydowiellaceae*), which causes petiole blight and root rot of strawberry and is distinct from the above taxa. An updated multilocus phylogeny for the *Diaporthales* is provided with representatives of currently known families.

## INTRODUCTION

The order *Diaporthales* is one of the largest and best-defined orders in the *Sordariomycetes* (Castlebury et al. 2002; Zhang et al. 2006; Rossman et al. 2007). The order comprises many destructive plant pathogens causing diseases on various crops, ornamental plants and forest trees, as well as numerous endophytic and saprobic fungal species (Udayanga et al. 2011, 2015; Shuttleworth and Guest 2017; Senanayake et al. 2017a; Jiang et al. 2019, 2020). It currently contains approximately 31 families supported by molecular data, with many recent additions and segregations of genera and families within the order (Castlebury et al. 2002; Lumbsch and Huhndorf 2007; Rossman et al. 2007, 2015, 2016; Yang et al. 2018; Voglmayr et al. 2017, 2019a; Jiang et al. 2020). Although the phylogenetic relationships and species composition of the majority of commonly encountered pathogenic genera are known, much work remains to be done concerning more obscure taxa from various geographic locations around the world (Zhang and Blackwell 2001; Rossman et al. 2007; Yun and Rossman 2011; Crous et al. 2012a, 2012b; Walker et al. 2012; Rossman et al. 2015).

The genus *Fragaria*, better known as strawberry, belongs in the plant family *Rosaceae* and is well known for its edible fruits (Hancock 1999). Worldwide, there are more than 25 described species, including wild species and many hybrids and cultivars (Potter et al. 2000; Staudt 2009; Zhong et al. 2018). The modern cultivated/garden strawberry, *Fragaria* × *ananassa* (Weston) Duchesne ex Rozier is one of the most important economic fruit crops worldwide (Simpson 2018). Pre- and post-harvest fungal diseases caused by various pathogens have a great impact on strawberry production, decreasing subsequent fruit yield and quality (Maas 1998; Koike et al. 2009; Xu et al. 2015; Baroncelli et al. 2015; Abdelfattah et al. 2016). Among those pathogenic fungi, three of the destructive species namely *Gnomoniopsis fructicola*, *Paragnomonia fragariae*, and *Phomopsis obscurans* are members of the order *Diaporthales* (*Sordariomycetes*, *Ascomycota*).

Among various plant pathogens, *Phomopsis obscurans* is known to cause leaf blight and fruit rot in most of the strawberry growing regions of the world (Plakidas 1964; Sutton 1965; Eshenaur and Milholland 1989; Maas 1998; Ellis et al. 2000; Udayanga et al. 2011). Due to the implementation of one name for pleomorphic fungi, all species formerly known as *Phomopsis* and phylogenetically congeneric should now be placed in the genus *Diaporthe* (Udayanga et al. 2012, 2014a, 2014b; Rossman et al. 2014; Gomes et al. 2013; Fan et al. 2018). However, the generic placement of the strawberry leaf blight fungus has always been subject to uncertainty.

Ellis and Everhart (1894) formally described the species causing leaf blight of *Fragaria* as *Phoma obscurans* based on a collection from West Virginia (USA). The fungus was reported from various regions of North America in subsequent studies. A severe outbreak of leaf blight was reported from Indiana in 1919 (Anderson 1920; Plakidas 1964). The causal agent of this outbreak was identified by Anderson (1920) as *Dendrophoma obscurans*. Sutton (1965) revisited the concept of *Dendrophoma* and suggested *D. obscurans* was not congeneric with the type species, *D. cytisporoides.* The type species of *Dendrophoma*, *D*. *cytosporoides*, belongs to the family *Chaetosphaeriaceae* (*Chaetosphaeriales*) based on available molecular data (Crous et al. 2012a). *Phoma obscurans* has been also known as *Sphaeropsis obscurans* and *Phyllosticta obscurans* in taxonomic literature (Kuntze 1898; Tassi 1902). However, the taxonomic affinity of *P. obscurans* to either *Sphaeropsis* or *Phyllostica* was unknown. Based on comparisons with representative *Phomopsis* species, the name *Phomopsis obscurans* was proposed for the leaf blight fungus, by Sutton (1965).

In 1916, *Sphaeronaemella fragariae* was reported to be the causal agent of “*Sphaeronaemella*” rot in strawberry (Stevens and Peterson 1916; Maas 1998). This species was not accepted in the mycoparasitic genus *Sphaeronaemella* by Malloch (1974), as a sexual morph was not known (Stevens and Peterson 1916). Hausner and Reid (2004) utilized nuc 18S rDNA sequence data of the ex-syntype isolate (CBS 118.16) of *S. fragariae* and confirmed it did not group with the type species of *Sphaeronaemella*, *S. helvellae*. Therefore, they considered it to be a synonym of *Phomopsis obscurans* in the *Diaporthales*. In the study by Senanayake et al. (2017a), the name *Microascospora fragariae* was proposed, based on *S. fragariae* and unauthenticated ITS sequences from an unpublished study. However, the name *Phoma obscurans* has since been found to be the oldest name for this fungus.

Similarly, confusion has existed among *Gnomonia*-like species associated with strawberry (Sogonov et al. 2008; Walker et al. 2010). The name *Gnomonia comari* is commonly used in older literature to refer to the fungus causing leaf blotch and fruit rot of strawberry. However, Sogonov et al. (2008) expanded the concept of *Gnomoniopsis* (*Gnomoniaceae*) to include *G. comari* as *Gnomoniopsis comari*. That same study revealed *G. comari* to be distinct from the causal agent of leaf blotch and petiole blight of strawberry in Europe and North America, known as *Gnomoniopsis fructicola.* Therefore, *G. comari* is now considered to be associated exclusively with *Comarum palustre* and not as a pathogen of strawberry*.*

The third diaporthalean fungus associated with strawberry, *Paragnomonia fragariae*, is known to cause petiole blight and root rot of perennial strawberry in Northern Europe and has been shown to be not congeneric with *Gnomonia* (*Gnomoniaceae*) based on molecular data (Moročko and Fatehi 2007)*.* Morphologically, this species is similar to gnomoniaceous taxa with an apparently limited distribution in Europe and no known asexual morph (Moročko 2006; Moročko and Fatehi 2007). Recently, Moročko-Bičevska et al. (2019) lectotypified it based on illustrations from the original description, providing taxonomic and nomenclatural clarifications, and designating an epitype specimen from Latvia,

The aims of this study were to infer the evolutionary relationships and revise the taxonomy of diaporthalean fungi associated with strawberry utilizing fresh collections, ex-type isolates and preserved fungal specimens from herbaria. An updated multilocus phylogeny of the order diaporthales, including fungal isolates from strawberry and modern taxonomic descriptions and illustrations are provided for the fungi reassessed in this study.

## MATERIALS AND METHODS

### Sample sources and morphology

Strains of pathogenic fungi causing leaf blight of strawberry (*Phomopsis obscurans)* were obtained from a conventionally managed, matted-row production system at a private farm in Germantown, MD, USA (Black et al. 2002). In addition, samples were collected from two locations at the Beltsville Agriculture Research Center (USDA-ARS) in Beltsville, MD, where neither fumigants nor fungicides had been used: the Student Discovery Garden and yield-trial plots for the strawberry breeding program (Lewers et al. 2019). Pure cultures of the pathogens were isolated by single spore isolation (Udayanga et al. 2012) from leaf specimens with typical mature disease symptoms. Other fresh specimens and pure cultures were obtained from culture collections and various contributors (Table 1). Holotype and other specimens were obtained from the United States National Fungus Collections (BPI) and other herbaria.

**Table 1:** Isolates and DNA sequences used in this study

Families in Diaporthales	Culture collection/Isolate	Species	Host	Country	GenBank Accessions			
					LSU	ITS	RPB2	TEF1
Apiosporopsidaceae	CBS 771.79	*Apiosporopsis carpinea*	*Carpinus betulus*	Switzerland	AF277130	–	–	–
Apoharknessiaceae	CBS 111377^*^	*Apoharknessia insueta*	*Eucalyptus pellita*	Brazil	AY720814	JQ706083	–	MN271820
–	CBS 114575	*Apoharknessia insueta*	*Eucalyptus* sp.	Colombia	MN172370	MN172402	–	MN271821
Asterosporiaceae	MFLU 15–3555	*Asterosporium asterospoermum*	*Fagus sylvatica*	Italy	MF190062	–	–	–
Auratiopycnidiellaceae	CBS 132180^*^/ CPC 16371	*Auratiopycnidiella tristaniopsis*	*Tristaniopsis laurina*	Australia: New South Wales	JQ685522	JQ685516	–	MN271825
–	CPC 16371	*Auratiopycnidiella tristaniopsis*	*Tristaniopsis laurina*	Australia: New South Wales	MN172374	MN172405	–	MN271826
Coryneaceae	D201	*Coryneum umbonatum*	*Quercus robur*	Austria	MH674329	MH674329	MH674333	MH674337
–	CFCC 52319/isolate 89–1^*^	*Coryneum gigasporum*	*Castanea mollissima*	China	MH683557	MH683565	–	–
Cryphonectriaceae (subclade1)	ATCC 38755	*Cryphonectria parasitica*	*Castanea dentata*	USA	NG_027589	AY141856	DQ862017	EU222014
–	ATCC 48198/CMW7048	*Cryphonectria parasitica*	*Quercus virginiana*	USA	JN940858	JN942325	–	–
–	CFCC 52150	*Cryphonectria parasitica*	*Castanea mollissima*	China	MH514021	MG866018	–	MN271848
Cryphonectriaceae (subclade2)	CBS 112916^*^/CMW62/CRY-98	*Chrysoporthe australafricana*	*Eucalyptus grandis*	South Africa	AY194097	AF292041	–	MN271832
–	CBS 118654^*^	*Chrysoporthe cubensis*	*Eucalyptus grandis*	Cuba	MN172378	DQ368773	–	MN271834
Cytosporaceae	CFCC 89982^*^	*Cytospora chrysosperma*	*Ulmus pumila*	China	KP310805	KP281261	KU710952	KP310848
–	CFCC 89633	*Cytospora eleagni*	*Elaeagnus angustifolia*	China	KF765693	KF765677	KU710956	KU710919
–	CBS 202.36^*^	*Cytospora viridistroma*	*Cercis canadensis*	Georgia	MN172388	MN172408	–	MN271853
Diaporthaceae	AR 3405^*^/CBS 135422	*Diaporthe citri*	*Citrus* sp.	USA	**MT378365**	KC843311	**MT383081**	KC843071
–	AR 4855	*Diaporthe novem*	*Lactuca muralis*	France	**MT378366**	**MT378351**	**MT383082**	**MT383100**
–	CBS 592.81^*^	*Diaporthe helianthi*	*Helianthus annuus*	Serbia	**MT378370**	NR_103698	–	KC343841
–	CBS 138594/AR 5193^*^	*Diaporthe eres*	*Ulmus* sp.	Germany	**MT378367**	KJ210529	**MT383083**	KJ210550
–	CBS 125529/AR 4658	*Mazzantia galii*	*Galium aparine*	France	MH875041	MH863563	–	**MT383101**
Diaporthosporellaceae	CBS 140348^*^	*Diaporthella cryptica*	*Corylus avellana*	Italy	MN172390	MN172409	MN271800	MN271854
–	CFCC 51994^*^	*Diaporthosporella cercidicola*	*Cercis chinensis*	China	KY852515	KY852492	–	MN271855
Diaporthostomataceae	CFCC 52101	*Diaporthostoma machili*	*Machilus leptophylla*	China	MG682021	MG682081	MG682041	MG682061
–	CFCC 52100^*^	*Diaporthostoma machili*	*Machilus leptophylla*	China	MG682020	MG682080	MG682040	MG682060
Dwiroopaceae	CBS 109755^*^	*Dwiroopa lythri*	*Lythrum salicaria*	USA	MN172389	MN172410	MN271801	MN271859
–	CBS 143163^*^	*Dwiroopa punicae*	*Punica granatum* var. *azadi*	USA:Minnesota	MK510686	MK510676	MK510692	–
Erythrogloeaceae	CBS 132185^*^/CPC 18819	*Erythrogloeum hymenaeae*	*Hymenaea courbaril*	Brazil	JQ685525	JQ685519	–	–
–	CFCC 52106^*^	*Dendrostoma osmanthi*	*Osmanthus fragrans*	China	MG682013	MG682073	MG682033	MG682053
Folicryphiaceae	CFCC 53025^*^	*Neocryphonectria chinensis*	*Carpinus turczaninowii*	China	MN172397	MN172414	MN271812	MN271893
–	CFCC 53027^*^/CFCC 53027	*Neocryphonectria carpini*	*Carpinus turczaninowi*	China	MN172396	MN172413	–	–
Gnomoniaceae	DMW 108/CBS 128442	*Ophiognomonia rosae*	*Fragaria vesca*	USA	**MT378355**	JF514851	**MT383086**	JF514824
–	CBS 851.79	*Ophiognomonia rosae*	*Comarum palustre*	Finland	**MT378356**	EU254930	**MT383071**	JQ414153
–	CBS 121226/AR4275^*^	*Gnomoniopsis fragariae*	*Fragaria vesca*	USA	EU255115	EU254824	EU219250	EU221961
–	DMW 63	*Gnomoniopsis fragariae*	*Fragaria* × *ananassa*	USA	**MT378357**	**MT378343**	**MT383072**	**MT383089**
–	DMW 61	*Gnomoniopsis fragariae*	*Fragaria* sp.	USA	**MT378358**	**MT378344**	**MT383073**	**MT383090**
–	VPRI 15547	*Gnomoniopsis fragariae*	*Fragaria* × *ananassa*	Australia	**MT378359**	**MT378345**	**MT383087**	**MT383091**
–	CBS 275.51/ATCC 11430	*Gnomoniopsis fragariae*	*Fragaria* sp.	Canada:Ontario	MH868373	EU254829	**MT383088**	**MT383092**
–	CBS 208.34	*Gnomoniopsis fragariae*	*Fragaria* sp.	France	EU255116	EU254826	EU219284	EU221968
–	CBS 904.79	*Gnomoniopsis tormentillae*	*Potentilla erecta*	Switzerland	EU255133	EU254856	–	GU320795
–	CBS 806.79	*Gnomoniopsis comari*	*Comarum palustre*	Finland	EU255114	EU254821	–	GU320810
Harknessiaceae	CBS 120033^*^/CFCC 53027	*Harknessia gibbosa*	*Eucalyptus delegatensis*	Tasmania	EF110615	EF110615	–	MN271868
–	CBS 120030^*^	*Harknessia ipereniae*	*Eucalyptus* sp.	Western Australia	EF110614	EF110614	–	MN271870
Juglanconidaceae	MAFF 410216	*Juglanconis oblonga*	*Juglans ailanthifolia*	Japan	KY427153	KY427153	KY427203	KY427222
–	CBS 121083	*Juglanconis juglandina*	*Juglans regia*	Austria	KY427148	KY427148	KY427198	KY427217
–	MAFF 410079^*^	*Juglanconis pterocaryae*	*Pterocarya rhoifolia*	Japan	KY427155	KY427155	KY427205	KY427224
Lamproconiaceae	MFLUCC 15–0870	*Lamproconium desmazieri*	*Tilia tomentosa*	Russia	KX430135	KX430134	MF377605	MF377591
–	MFLUCC 15–0872	*Lamproconium desmazieri*	*Tilia cordata*	Russia	KX430139	KX430138	–	MF377593
Macrohilaceae	CBS 140063^*^	*Macrohilum eucalypti*	*Eucalyptus piperita*	Australia	NG_058183	NR_154184	MN271810	–
–	CPC 10945	*Macrohilum eucalypti*	*Eucalyptus* sp.	New Zealand	DQ195793	DQ195781	–	–
Mastigosporellaceae	VIC44383^*^/COAD 2370	*Mastigosporella pigmentata*	*Qualea parviflora*	Brazil	MG587928	MG587929	–	–
–	CBS 136421^*^	*Mastigosporella anisophylleae*	*Anisophyllea* sp.	Zambia	KF777221	KF779492	–	MN271892
Melanconidaceae	CFCC 50474	*Melanconis itoana*	*Betula albosinensis*	China	KT732974	KT732955	KT732987	KT733004
–	CFCC 50475^*^	*Melanconis stilbostoma*	*Betula platyphylla*	China	KT732975	KT732956	KT732988	KT733005
–	CFCC 50471	*Melanconis betulae*	*Betula albosinensis*	China	KT732971	KT732952	KT732984	KT733001
Melanconiellaceae	AU01	*Greeneria uvicola*	*Vitis vinifera*	Australia	JN547720	–	–	–
–	OH35	*Greeneria uvicola*	*Vitis labrusca*	Ohio	AF362570	–	–	–
–	AR 3457	*Melanconiella spodiaea*	*Carpinus betulus*	Austria	AF408369	**MT378352**	**MT383074**	**MT383093**
–	AR 3462	*Melanconiella spodiaea*	*Carpinus betulus*	Austria	AF408370	**MT378353**	**MT383075**	**MT383094**
–	AR 3830/CBS 131494	*Melanconiella elegans*	*Carpinus caroliniana*	USA	JQ926264	JQ926264	JQ926335	JQ926401
–	CBS 125597	*Melanconiella chrysodiscosporina*	*Carpinus betulus*	Austria	MH875191	MH863730	–	–
–	BPI 878343	*Melanconiella ellisii*	*Carpinus caroliniana*	USA	JQ926271	JQ926271	JQ926339	JQ926406
–	MFLU 15–1112^*^	*Microascospora rubi*	*Rubus ulmifolius*	Italy	MF190099	MF190154	MF377611	MF377582
–	MFLU 17–0883	*Microascospora rubi*	*Rubus ulmifolius*	Italy	MF190098	MF190153	–	MF377581
–	M1261/DS016	*Paraphomopsis obscurans*	*Fragaria* × *ananassa*	USA	**MT378360**	**MT378346**	**MT383076**	**MT383095**
–	CBS 143829/M1262/DS020^*^	*Paraphomopsis obscurans*	*Fragaria* × *ananassa*	USA	**MT378361**	**MT378347**	**MT383077**	**MT383096**
–	M1259/DS013	*Paraphomopsis obscurans*	*Fragaria* × *ananassa*	USA	**MT378362**	**MT378348**	**MT383078**	**MT383097**
–	M1333/DS133	*Paraphomopsis obscurans*	*Fragaria* × *ananassa*	USA	**MT378363**	**MT378349**	**MT383079**	**MT383098**
–	M1278/DS055	*Paraphomopsis obscurans*	*Fragaria* × *ananassa*	USA	**MT378364**	**MT378350**	**MT383080**	**MT383099**
–	strain 1–1	*Paraphomopsis obscurans (as. Sphaeronaemella fragariae)*	*Fragaria* sp.	China	–	HM854850	–	–
–	strain 1–3	*Paraphomopsis obscurans (as. Sphaeronaemella fragariae)*	*Fragaria* sp.	China	–	HM854852	–	–
–	strain 12	*Paraphomopsis obscurans (as. Sphaeronaemella fragariae)*	*Fragaria* sp.	China	–	HM854849	–	–
Phaeoappendicosporaceae	MFLUCC 13–0161^*^/MFLU 12–2131	*Phaeoappendicospora thailandensis*	*Quercus* sp.	Italy	MF190102	MF190157	–	–
Prosopidicolaceae	CBS 113529^*^	*Prosopidicola mexicana*	*Prosopis glandulosa*	USA	KX228354	AY720709	–	–
–	CBS 141298/CPC 27478^*^	*Prosopidiocola albizziae*	*Albizzia falcataria*	Malaysia	KX228325	KX228274	–	–
Pseudomelanconidaceae	CFCC 52787^*^	*Neopseudomelanconis castaneae*	*Castanea mollissima*	China	MH469164	MH469162	–	–
–	CFCC 52110^*^	*Pseudomelanconis caryae*	*Carya cathayensis*	China	MG682022	MG682082	MG682042	MG682062
Pseudoplagiostomaceae	CBS 115722/CMW 6674	*Pseudoplagiostoma oldii*	*Eucalyptus camaldulensis*	Australia	GU973610	GU973535	–	GU973565
–	CPC 14161	*Pseudoplagiostoma eucalypti*	*Eucalyptus camaldulensis*	Vietnam	GU973604	GU973510	–	GU973540
Schizoparmaceae	CBS 112640^*^/STE-U 3904	*Coniella eucalyptorum*	*Eucalyptus grandi*s × *tereticornis*	Queensland	AY339290	AY339338	KX833452	KX833637
–	CBS 110394^*^	*Coneilla peruensis*	soil in rain forest	Peru	KJ710441	KJ710463	KX833499	KX833695
Stilbosporaceae	CBS 122529^*^	*Stilbospora longicornuta*	*Carpinus betulus*	Austria	KF570164	KF570164	KF570194	KF570232
–	CBS 117025^*^	*Stegonsporium acerophilum*	*Acer saccharum*	USA: Tennessee	EU039993	EU039982	KF570173	EU040027
Sydowiellaceae	AR 3809	*Chapeckia nigrospora*	*Betula* sp.	USA	EU683068	–	–	–
–	F129/P3/1^*^	*Paragnomonia fragariae*	*Fragaria* × *ananassa*	Latvia	MK524447	MK524430	–	MK524466
–	GF300/M1530	*Paragnomonia fragariae*	*Fragaria* sp.	France	**MT378368**	–	**MT383084**	**MT383102**
–	GF301/M1531	*Paragnomonia fragariae*	*Fragaria* sp.	France	**MT378369**	–	**MT383085**	**MT383103**
–	MFLU 16–2864^*^	*Sillia karstenii*	*Centaurea* sp.	Italy	KY523500	KY523482	KY501636	–
Synnemasporellaceae	CFCC 52094	*Synnemasporella aculeans*	*Rhus chinensis*	China	MG682026	MG682086	MG682046	MG682066
–	CFCC 52097^*^	*Synnemasporella toxicodendri*	Toxicodendron sylvestre	China	MG682029	MG682089	MG682049	MG682069
Tirisporellaceae	BCC 00018	*Thailandiomyces bisetulosus*	*Licuala longicalycata*	Thailand	EF622230	–	–	–
–	BCC 38312	*Tirisporella beccariana*	*Nypa fruticans*	Thailand	JQ655449	–	–	–
Tubakiaceae	CBS 129012^*^	*Tubakia iowensis*	*Quercus macrocarpa*	USA	MG591971	JF704194	–	MG603576
–	CBS 127490^*^	*Tubakia seoraksanensis*	*Quercus mongolica*	South Korea	KP260499	MG591907	–	MG592094
–	CBS 114386	*Tubakia dryina*	*Quercus robur*	New Zealand	JF704188	MG591852	–	MG592040
–	CPC 13806	*Racheliella wingfieldiana*	*Syzygium guineense*	South Africa	MG592006	MG591911	MG976487	MG592100
–	CBS 189.71^*^	*Oblongisporothyrium castanopsidis*	*Castanopsis cuspidata*	Japan	MG591943	MG591850	–	MG592038
–	CBS 124732	*Oblongisporothyrium castanopsidis*	*Castanopsis cuspidata*	Japan	MG591942	MG591849	MG976453	MG592037
–	MUCC 2293^*^	*Paratubakia subglobosoides*	*Quercus glauca*	Japan	MG592010	MG591915	MG976491	MG592104
–	CBS 193.71^*^	*Paratubakia subglobosa*	*Quercus glauca*	Japan	MG592009	MG591914	MG976490	MG592103
–	CPC 31361	*Sphaerosporithyrium mexicanum*	*Quercus eduardii*	Mexico	MG591988	MG591894	–	MG592081
–	CPC 33021^*^	*Sphaerosporithyrium mexicanum*	*Quercus eduardii*	Mexico	MG591990	MG591896	MG976473	MG592083
–	MUCC 2304^*^	*Involutiscutellula rubra*	*Quercus phillyraeoides*	Japan	MG591995	MG591901	MG976478	MG592088
–	CBS 192.71^*^	*Involutiscutellula rubra*	*Quercus phillyraeoides*	Japan	MG591993	MG591899	MG976476	MG592086
Outgroup (Magnaporthales)	MFLU 18–2323^*^/MFLUCC 18–1337	*Ceratosphaeria aquatica*	submerged wood	China	MK835812	MK828612	MN156509	MN194065
–	CG-4/M83^*^	*Pyricularia grisea*	*Digitaria* sp.	USA	JX134683	JX134671	–	JX134697

Abbreviations of the culture collections: ATCC: American Type Culture collection; CMW:FABI fungal culture collection; CBS:CBS-KNAW culture collection, Westerdijk Fungal Biodiversity Institute; MFLU: Mae Fah Luang University Herbarium; MFLUCC: Mae Fah Luang University Culture Collection; CFCC: China Forestry Culture Collection Center; STE-U: culture collection of the Department of Plant Pathology at the University of Stellenbosch; AR, M, DMW: Cultures housed at MNGDBL, USDA-ARS, Beltsville, Maryland; CPC: Culture collection of Pedro Crous, housed at Westerdijk Fungal Biodiversity Institute; MUCC: Murdoch University Culture Collection; BCC: BIOTEC Culture Collection, Bangkok, Thailand; VPRI: Victoria Plant Pathology Herbarium. *Ex-type/epitype/neotype cultures or specimens are indicated by asterisks. Newly generated sequences in this study are bold

Morphological descriptions were based on pycnidia or perithecia formed on inoculated alfalfa stems placed on 2% water agar (WA), as well as from type specimens. Digital images of fruiting bodies were captured using a Discovery V20 stereomicroscope and AxioCam HrC digital camera (Carl Zeiss Microscopy, Thornwood, New York, USA) imaging system. Whenever possible, 20–30 measurements were made of the structures mounted in 5% KOH using a Carl Zeiss Axioplan2 compound light microscope. The sample sizes are given in parentheses with mean and standard deviation. Triplicates of the cultures for each isolate were used for determining colony characters on Potato Dextrose Agar (PDA), Malt Extract Agar (MEA, Becton, Dickinson and Company, Franklin Lakes, NJ, USA), and V8 juice agar (V8A) (Dhingra and Sinclair 1985) at 25 °C in indoor light. After 1 wk., and color of the colonies were recorded. The colony color codes are given within the parenthesis according to the charts by Rayner (1970). For determination of growth rates, triplicate PDA plates were inoculated with 5 mm in diam plugs of actively growing fungal cultures. Mycelial growth was measured daily along two perpendicular lines drawn at the center of the colonies and continued for two weeks. Radial growth rates were calculated and expressed in mm day^− 1^. Digital images were captured and cultural characteristics were observed as described in Udayanga et al. (2014a, 2014b).

### DNA extraction, PCR and sequencing

Mycelial scrapings (50–60 mg) from the leading edge of cultures on PDA, incubated for 4–5 d at 25 °C were harvested and lysed in tubes containing 500 μm garnet media and a 6mm zirconium bead (OPS Diagnostics, Lebanon, New Jersey) with the Fast Prep FP120 benchtop bead-beating instrument (Thermo Fischer Scientific Inc., Waltham, Massachusetts) for 60 s (20 s × 3 with 10 s intervals). Genomic DNA was extracted with the DNeasy Plant Mini Kit (Qiagen, Inc., Valencia, California) according to the manufacturer’s instructions. DNA was eluted from DNAeasy mini spin column using 50 μl of elution buffer and visualized with agarose gel electrophoresis in 1% agarose gels stained with SYBR Safe DNA Gel Stain (Invitrogen, Eugene, Oregon).

The nuc rDNA internal transcribed spacer ITS1–5.8S-ITS2 region (*ITS*), nuc 28S rRNA gene (*LSU*), translation elongation factor 1-alpha (*TEF1)* and second largest subunit of RNA polymerase II (*RPB2)* gene regions were amplified on a Bio-Rad Dyad Peltier thermal cycler in a 25 μL reaction volume: 10–15 ng genomic DNA, 12.5 μL Quick load Taq 2x Master Mix (New England BioLabs, Ipswich, Massachusetts), 1 μL 10 mM of each primer, and nuclease-free water to adjust volumes to 25 μL. Amplification and DNA sequencing of *ITS* region were performed using forward and reverse primer pair ITS1 and ITS4 (White et al. 1990), as described by Udayanga et al. (2014a). Amplification of 28S ribosomal DNA region was performed using the forward and reverse primer pair LROR and LR7 (Vilgalys and Hester 1990), under the following conditions: 95 °C 5 min, (95 °C: 60 s, 55 °C: 60 s, 72 °C: 60 s) × 39 cycles, 72 °C 10 min. DNA sequencing was performed using the same PCR primers with additional internal primers LR3R and LR5 (Rehner and Samuels 1995). The *RPB2* gene region was amplified using the forward and reverse primer pair, fRPB2-5F and fRPB2-7cR (Liu et al. 1999) under the following conditions: 95 °C 5 min, 95 °C 1 min, [55 °C 2 min - increase 0.2 °C per second until 72 °C (slow ramp), 72 °C 2 min] × 34 cycles, 72 °C 10 min and sequenced using the same primers. The *TEF1* region was amplified and sequenced using the primer pair EF728f (Carbone and Kohn (1999) and EF2 (Rehner (2001), using a modified touchdown PCR protocol: 95 °C 5 min, [95 °C: 30 s, 66 °C: 30 s decrease 1 °C in every cycle, 72 °C: 80 s cycle to step 2] × 10 cycles [95 °C: 30 s, 56 °C 30 s, 72 °C 80 s] × 40 cycles, 72 °C 10 min.

PCR products were visualized as above. Excess primers and dNTPs were removed from PCR mixtures with ExoSAP-IT (USB Corp., Cleveland, Ohio) according to the manufacturer’s instructions. Amplicons were sequenced using the BigDye Terminator v. 3.1 cycle sequencing kit (Life Technologies, Grand Island, New York) on an Applied Biosystems 3130xl Genetic Analyzer (Thermo Fisher Scientific, Waltham, Massachusetts).

### Sequence alignment and phylogenetic analyses

The newly generated raw sequences were assembled into contigs with Sequencher 5.0 for Windows (Gene Codes Corp., Ann Arbor, Michigan). Additional sequences were obtained from GenBank, including ex-type or other reference sequences (Table 1). All sequence conversions and manual alignments were performed in Bioedit v.7.2.5 (Hall 1999) and CLC Sequence Viewer 7.7 (http://www.clcbio.com/products/clc-sequence-viewer/). Sequences were aligned with MAFFT v.7 using Auto (FFT-NS-1, FFT-NS-2, FFT-NS-i or L-INS-i depending on data size) strategy (http://mafft.cbrc.jp/alignment/server/) (Katoh and Standley 2013).

Isolates were selected to represent each of the 31 known families in the *Diaporthales* based on the latest available literature. Each taxon selected was represented by at least an LSU sequence. In addition to fungal isolates from *Fragaria*, several new sequences were generated for representative taxa of the order: *Ophiognomonia rosae* (DMW 108, CBS 851.79), *Melanconiella spodiaea* (AR 3457, AR 3462), *Diaporthe eres* (AR 5193), *D. novem* (AR 4855), *D. citri* (AR 3405), *D. helianthi* (CBS 592.81) and *Mazzantia galii* (AR 4658). Two taxa in the *Magnaporthales* (*Sordariomycetes*), *Pyricularia grisea* (M83) and *Ceratosphaeria aquatica* (MFLU18–2323), were used as outgroup taxa in the phylogenetic analyses.

Phylogenetic reconstructions of concatenated and individual gene regions were performed using Maximum Likelihood (ML) and Bayesian Inference (BI) (Felsenstein 1981; Huelsenbeck et al. 2001). Individual datasets were tested for congruency using the 70% reciprocal bootstrap (BS) threshold method as described by Gueidan et al. (2007). ML gene trees were estimated using the software RAxML 8.2.8 Black Box (Stamatakis 2006; Stamatakis et al. 2008) in the CIPRES Science Gateway platform (Miller et al. 2010). For the concatenated dataset, all free-modal parameters were estimated by RAxML with an ML estimate of 25 per site rate categories. The concatenated dataset was partitioned by locus and the gaps were treated as missing data. The RAxML analysis utilized the GTRCAT model of nucleotide substitution with the additional options of modeling rate heterogeneity (G) and proportion invariable sites (I).

Bayesian analysis was performed using MrBayes v. 3.1.2 (Huelsenbeck and Ronquist 2001) and substitution models were determined in MrModeltest v. 2.3 (Nylander 2004). Bayesian reconstructions were performed using MrBayes 3.1.2. Six simultaneous Markov chains were run for 1,000,000 generations and trees were sampled every 100 generations, resulting in 10,000 total trees. The first 25% of the trees, representing the burn-in phase of the analyses, were discarded, and the remaining trees were used for calculating posterior probabilities (PP) in the majority rule consensus tree. Trees were visualized in FigTree v. 1.4.4 (Rambaut 2018). The DNA sequence alignments, single gene and combined trees were deposited in the USDA AgData Commons: 10.15482/USDA.ADC/1518737.

## RESULTS

### Phylogenetic analyses, limits and boundaries of genera and families

**Fig. 1:**
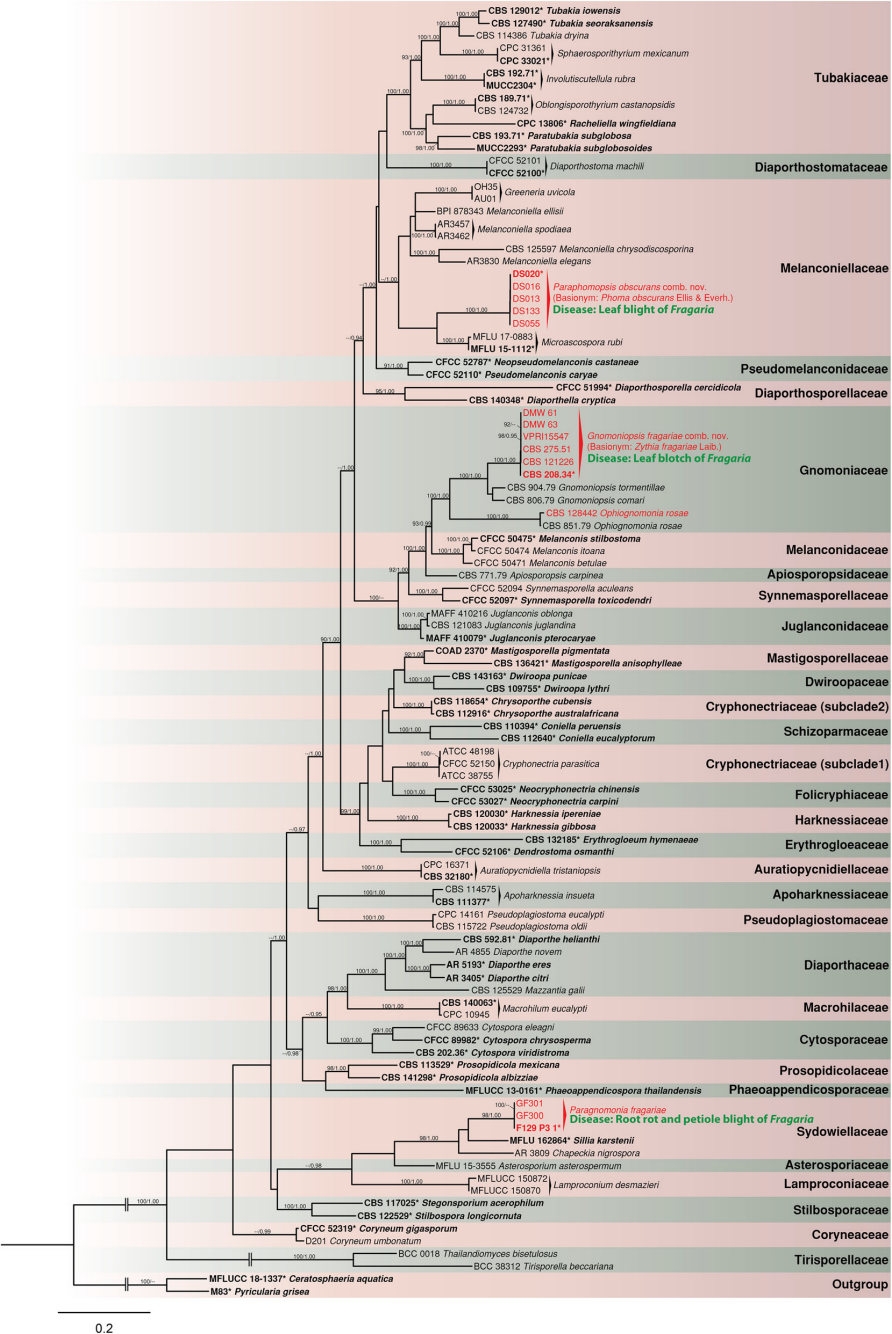
ML tree generated based on combined *LSU*, *ITS*, *RPB2*, and *TEF1* alignment of representative taxa in the order *Diaporthales.* Isolates from *Fragaria* are indicated in red. Ex-type/epitype isolates are in bold and marked with asterisk (*). The ML bootstrap values/Bayesian PP greater than 90% /0.90 are indicated above or below the respective branches. The tree is rooted with *Pyricularia grisea* (M83) and *Ceratosphaeria aquatica* (MFLU 18–2323) (*Magnaporthaceae*, *Magnaporthales*)

In total, 60 new DNA sequences were generated in this study. The approximate sizes of the target fragments of *ITS*, *LSU*, *RPB2* and *TEF1* were observed to be 600 bp, 1200 bp, 1000 bp and 650 bp, respectively. The remaining sequences were downloaded from GenBank (Table 1). Each gene region was aligned individually before concatenation in a sequence alignment consisting of 103 taxa representing 48 genera in 31 families of *Diaporthales*, including the isolates of fungi associated with *Fragaria* obtained in this study. The final combined four gene alignment consisted of 3899 total characters including gaps. Each taxon is represented by at least the *LSU* sequence. The ML tree resulting from the RAxML analysis had a final ML Optimization Likelihood of − 61,871.952114 and the following model parameters: alpha = 0.344711, pi(A) = 0.239113, pi(C) = 0.263167, pi(G) = 0.271106, and pi(T) = 0.226613. This tree was used to represent the phylogeny of the order *Diaporthales* (Fig. 1).

The phylogeny inferred from the combined analysis of four loci resolved deeper nodes where confusion has remained at familial and generic boundaries when using only LSU data or other single gene regions (trees available at 10.15482/USDA.ADC/1518737). Major monophyletic groups representing families and genera were resolved with well-supported branches. Both BI and ML trees resolved the 31 families and 48 genera including the new genus described herein.

Multilocus phylogeny generated in this study placed the *Fragaria* isolates in the *Melanconiellaceae*, *Sydowiellaceae* and *Gnomoniaceae*. Based on the combined analysis, we determined that the isolates from strawberry causing leaf blight known to date as *Phomopsis obscurans*, are distinct from their closest relatives classified in *Melanconiella, Microascospora* or *Greeneria*. Therefore, a new genus *Paraphomopsis* is described below to accommodate the species formerly known as *Phomopsis obscurans*. The combined analysis further revealed that *Paraphomopsis obscurans* appears to be a sister taxon to *Microascospora rubi*, the type species of *Microascospora*. However, in the *LSU* and *TEF1* single gene analyses, *Microascospora rubi* and *Paraphomopsis obscurans* were found to be non-monophyletic, and they were diverged based on *ITS* and *RPB2* single gene trees. The ML bootstrap and BPP values for the node that groups *Microascospora rubi* and *Paraphomopsis obscurans* in the combined analysis were 65% and 0.68, respectively (≤90%/0.90, not shown in Fig. 1). Therefore, the taxa were not considered to be congeneric based on combined phylogeny. The three representative *ITS* sequences (HM854850, HM854852, HM854849), used by Senanayake et al. (2017a) to propose the name *Microascopora fragariae* (synonymized under *Paraphomopsis obscurans* below) were not included in the analyses due to lack of *LSU* sequences for those isolates. However, the *ITS* sequences for these isolates were 100% identical with the isolates of *Paraphomopsis obscurans* generated for this study.

The leaf blotch pathogen of strawberry, *Gnomoniopsis fructicola,* is currently placed in *Gnomoniaceae* with new molecular data from multiple isolates. The genus *Gnomoniopsis* (including syn. *Sirococcus*) represents a basal lineage to the rest of the genera in *Gnomoniaceae,* which contains the genera *Gnomonia*, *Plagiostoma*, *Cryptodiaporthe*, *Apiognomonia*, *Discula*, *Cryptosporella*, *Ophiognomonia* and *Anisogramma*. However, to preserve the historical concept of the widely accepted family *Gnomoniaceae*, which includes the major non-stromatic lineages in the *Diaporthales*, it is considered as a diverse single taxonomic entity with the assumption that intermediate genera in this family remain to be discovered. The closest family *Melanconidaceae* is clearly distinct from *Gnomoniaceae*. The new sequences generated for the fresh collection of the petiole blight and root rot pathogen, placed it within the *Sydowiellaceae* and conspecific with the recently designated epitype of *Paragnomonia fragariae* (F129/P3/1) with high ML bootstrap and BPP.

### Taxonomy

Based on the molecular phylogenetic assessment of the order we introduce a new genus and combination to accommodate the strawberry leaf blight fungus, with lecto- and epitypification of the taxon. A new combination is introduced for *Gnomoniopsis fructicola*, with lecto- and epitypification providing a revision of synonyms. The remaining strawberry isolates collected in this study belonged to *Paragnomonia fragrariae*, for which we provide a description based on fresh collections from France.

**Fig. 2:**
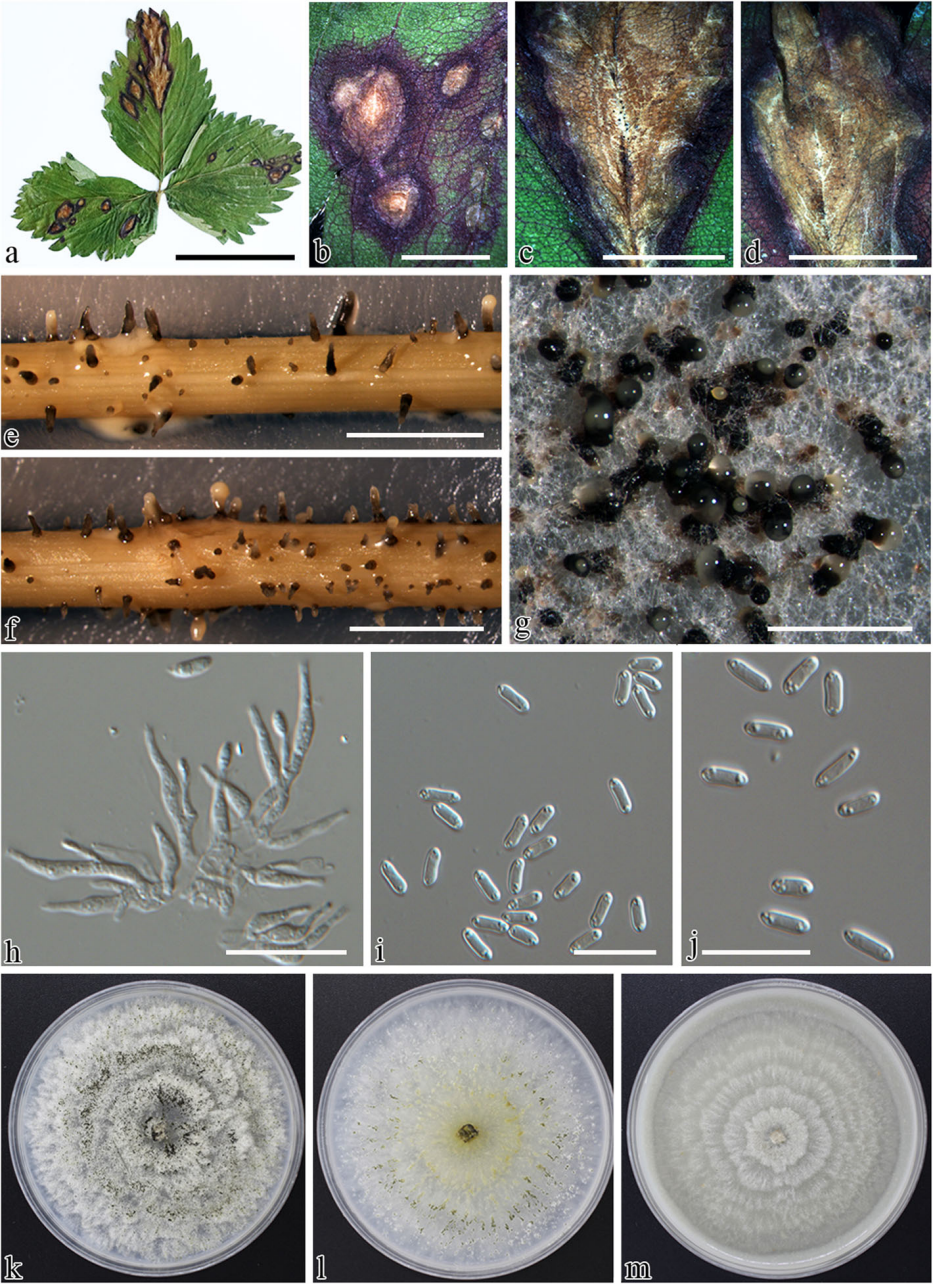
Morphology of *Paraphomopsis obscurans* (BPI 919201, culture CBS 143829/M1262, isolate DS020). **a** Infected leaf of *Fragaria × ananassa*. **b–d** Leaf blight symptoms under stereo microscope. **e,f** Pycnidia on alfalfa stems on WA. **g** Pycnidia on PDA. **h** Conidiophores. **i,j** Conidia. **k** 7-d-old culture on PDA. **l** 7-d-old culture on MEA. **m** 7-d-old culture on V8A. Scale bars: **a** = 4 cm, **b** = 1.5 cm, **c,d** = 1 cm, **e-g** = 300 μm, **h-j** = 10 μm

***Paraphomopsis*** Udayanga & Castl., **gen. nov**. Fig. 2.

MycoBank: MB 835529.

*Type species*: *Paraphomopsis obscurans* (Ellis & Everh.) Udayanga & Castl.

*Etymology*: Morphologically similar to the well-known asexual morph *Phomopsis* (curr. name *Diaporthe*), but phylogenetically distinct.

*Description: Asexual morph* coelomycetous*. Pycnidia* globose, ostiolate, embedded in tissue, erumpent at maturity, with a slightly elongated, black neck, wider towards the apex at maturity; walls parenchymatous, consisting of 3–4 layers of medium brown textura angularis. *Conidiophores* hyaline, smooth, branched, ampulliform, long, slender, wider at the base, *Conidiogenous cells* phialidic, cylindrical, terminal, slightly tapering towards apex, *alpha conidia* aseptate, hyaline, smooth, ellipsoidal to fusiform, often biguttulate, rarely multiguttulate with minute particles aggregated towards the ends, base subtruncate. *Sexual morph* unknown.

*Notes*: *Paraphomopsis* can be distinguished from its closely related genera (*Greeneria*, *Melanconiella, Microascopsora*) in *Melanconiellaceae* based on both molecular phylogeny and morphology. The genus *Paraphomopsis* is morphologically described herein, exclusively based on the characters of the asexual morph. The asexual morph of *Melanconiella* usually consists of both dark brown melanconium-like conidia as well as hyaline discosporina-like conidia (Voglmayr et al. 2012). Similarly, the genus *Greeneria*, which is typified by *G. uvicola*, forms pale brown conidia, smooth, variously shaped ranging from fusiform, oval, to ellipsoidal, each with a truncate base and obtuse to bluntly pointed apex (Farr et al. 2001). In *Paraphomopsis*, although the appearance of conidia is superficially similar to *Diaporthe* (*syn*. *Phomopsis*), micropscopic examination revealed that the shape and overall appearance are distinct from those in *Diaporthe* species. In general, conidia of *Paraphomopsis* are fusiform with minute guttules toward the end of the conidia, whereas most *Diaporthe* species form ovate to clavate conidia with no or prominent biguttulate or multiguttulate conidia. The morphology of sexual morph of the new genus described here remains unknown and is not available for comparison with other closely related genera. Although, the genus *Paraphomopsis* represents a sister clade to *Microascopora* in the phylogeny presented (Fig. 1), the asexual morph of the latter remains undetermined. The sexual morph of *Microascospora* distinct from other genera in the same family having immersed, solitary ascomata with narrow papilla with smaller hyaline, aseptate ascospores bearing long appendages (Senanayake et al. 2017a, 2017b). However, the sexual morph of the saprobic genus *Melanconiella* is identified by its inconspicuous ectostroma projecting above the substrate and the hyaline, yellow or brown ascopsores, with or without short, blunt appendages and occasionally with a thin gelatinous sheath (Voglmayr et al. 2012; Senanayake et al. 2017a, 2017b).

***Paraphomopsis obscurans*** (Ellis & Everh.) Udayanga & Castl. **comb. nov**. Fig. 2.

MycoBank: MB 835530.

*Basionym*: *Phoma obscurans* Ellis & Everh., Proc. Acad. Nat. Sci. Phil. 46: 357. 1894.

≡ *Sphaeropsis obscurans* (Ellis & Everh.) Kuntze, Revis. gen. pl. (Leipzig) 3(2): 1–576. 1898.

≡ *Phyllosticta obscurans* (Ellis & Everh.) Tassi, Bulletin Labor. Orto Bot. de R. Univ. Siena 5: 13. 1902.

≡ *Dendrophoma obscurans* (Ellis & Everh.) H.W. Anderson, University of Illinois Agricultural Experiment Station Bull. 229: 135. 1920.

≡ *Phomopsis obscurans* (Ellis & Everh.) B. Sutton, Trans. Br. Mycol. Soc. 48(4): 615. 1965.

= *Sphaeronaemella fragariae* F. Stevens & Peterson, Phytopathology 6: 258. 1916.

≡ *Microascospora fragariae* (F. Stevens & Peterson) Senan., Maharachch. & K.D. Hyde, Stud. Mycol. 86: 279. 2017.

*Type*: USA. WEST VIRGINIA: Fayette Co., on leaves of *Fragaria* sp., 08 July 1894, *Nutall LW (1600 (7620) (J.B. Ellis 554)),* (**Lectotype** designated here BPI 521547; MBT393834); ibid. (**Iso-lectotype** designated here, BPI 357247; MBT 393835); USA. MARYLAND: Beltsville Agriculture Research Center, Beltsville, on leaves of *Fragaria × ananassa*, 21 May 2015, *Udayanga D*. *DS020*, (**Epitype** designated here, BPI 919201; MBT 393833, ex-epitype culture M1262 = CBS 143829). GenBank: *ITS* = MT378347; *LSU* = MT378361; *TEF1* = MT383096; *RPB2* = MT383077.

*Description: Pycnidia* on alfalfa stems on WA: globose, ostiolate, scattered over the substrate, 40–55 μm diam, embedded in tissue, erumpent at maturity, with a slightly elongated, black neck 60–100 μm high, wider towards the apex at maturity, often with a yellowish, conidial cirrus extruding from ostiole; walls parenchymatous, consisting of 3–4 layers of medium brown textura angularis. *Conidiophores* hyaline, smooth, branched, ampulliform, long, slender, wider at the base, 9–12 μm long and wide. *Conidiogenous cells* phialidic, cylindrical, terminal, slightly tapering towards apex, 1.5–2.5 μm diam at the widest point. *Collarette* present and conspicuous. *Paraphyses* absent. *Alpha conidia* 5–7 × 1.5–2.2 μm (avg. ± SD = 6 ± 0.5 × 2 ± 0.2, *n* = 30), abundant in culture and on alfalfa stems, aseptate, hyaline, smooth, ellipsoidal to fusiform, often biguttulate and rarely multiple guttules and confined to minute particles clumped towards the vertices of the spore, base subtruncate. *Beta conidia* unknown.

*Culture* on PDA under artificial light at 25 °C for 1 wk., growth rate: 4.5 ± 0.2 mm/day (n = 3), white, sparse aerial mycelium, with pale olivaceous grey (120) pigmentation and abundant sporulation with aging, olivaceous grey (107) pigmentation developing in reverse.

*Additional specimens examined:* USA. MARYLAND: Beltsville Agriculture Research Center, Beltsville, on leaves of *Fragaria × ananassa*, 22 May 2015, *Udayanga D*. *DS013* (BPI 919179), living culture M1259; ibid, 19 June 2015, *Udayanga D*. *DS021*, June 082015 *DS134* (BPI 19204); ibid*, DS016* (BPI 919180), living culture M1261; ibid, Greenhouses at Beltsville Agriculture Research Centre, Beltsville, on leaves of *Fragaria × ananassa*, 29 Sept. 2015, *Udayanga D*. *GR002* (BPI 919182); ibid, Davis Mill Road, Germantown (Montgomery County), on leaves of *Fragaria* × *ananassa* ‘Darselect’, 24 June 2015, *Butler B*. DS053 (BPI 919185) living culture M1276; ibid, Davis Mill Road, Germantown (Montgomery County), on leaves of *Fragaria* × *ananassa* ‘Darselect’, 12 Oct. 2016, *Butler B. DS090* (BPI 919192).

*Geographic distribution:* Australia (Cook and Dubé 1989; Shivas 1989; Cunnington 2003), Brazil (Mendes et al. 1998), Brunei Darussalam (Peregrine and Bin Ahmad 1982), Bulgaria (Bobev 2009), China (Jinping 2011; Shi et al. 2013), Egypt (Haggag 2009; Abd-El-Kareem et al. 2019), Malawi (Peregrine and Siddiqi 1972), Myanmar (Thaung 2008), South Africa (Crous et al. 2000), Tonga (Dingley et al. 1981), USA: Florida, Maryland, North Carolina, Ohio, Oregon, Washington, West Virginia (Alfieri Jr et al. 1984; Cash 1953; Shaw 1973; Maas 1998; Farr and Rossman 2020).

*Notes:* Although the appearance of conidia is superficially similar to *Phomopsis* (*Syn*. *Diaporthe*), microscopic examination revealed that the shape and overall appearance of guttules are distinct from those in *Diaporthe* species. In general, conidia of *Paraphomopsis obscurans* are fusiform with minute guttules toward the end of the conidia, whereas most *Diaporthe* species bear ovate to clavate conidia with no or prominent biguttulate or multiguttulate conidia. *Paraphomopsis obscurans* can be distinguished from the closely related species *Microascospora rubi* and other genera in the family *Melanconiellaceae* based on its morphology and robust support of the multilocus phylogeny. Due to confusion of nomenclature and taxonomy, previous records of the pathogen from various geographic locations were linked to multiple names: *Phoma obscurans*, *Sphaeronaemella fragariae* and *Phomopsis obscurans*, or misidentified as *Gnomonia fragariae*, *Gnomonia comari* and *Gnomoniopsis fragariae.* Therefore, the actual distribution of the fungus may be largely underestimated.

**Fig. 3:**
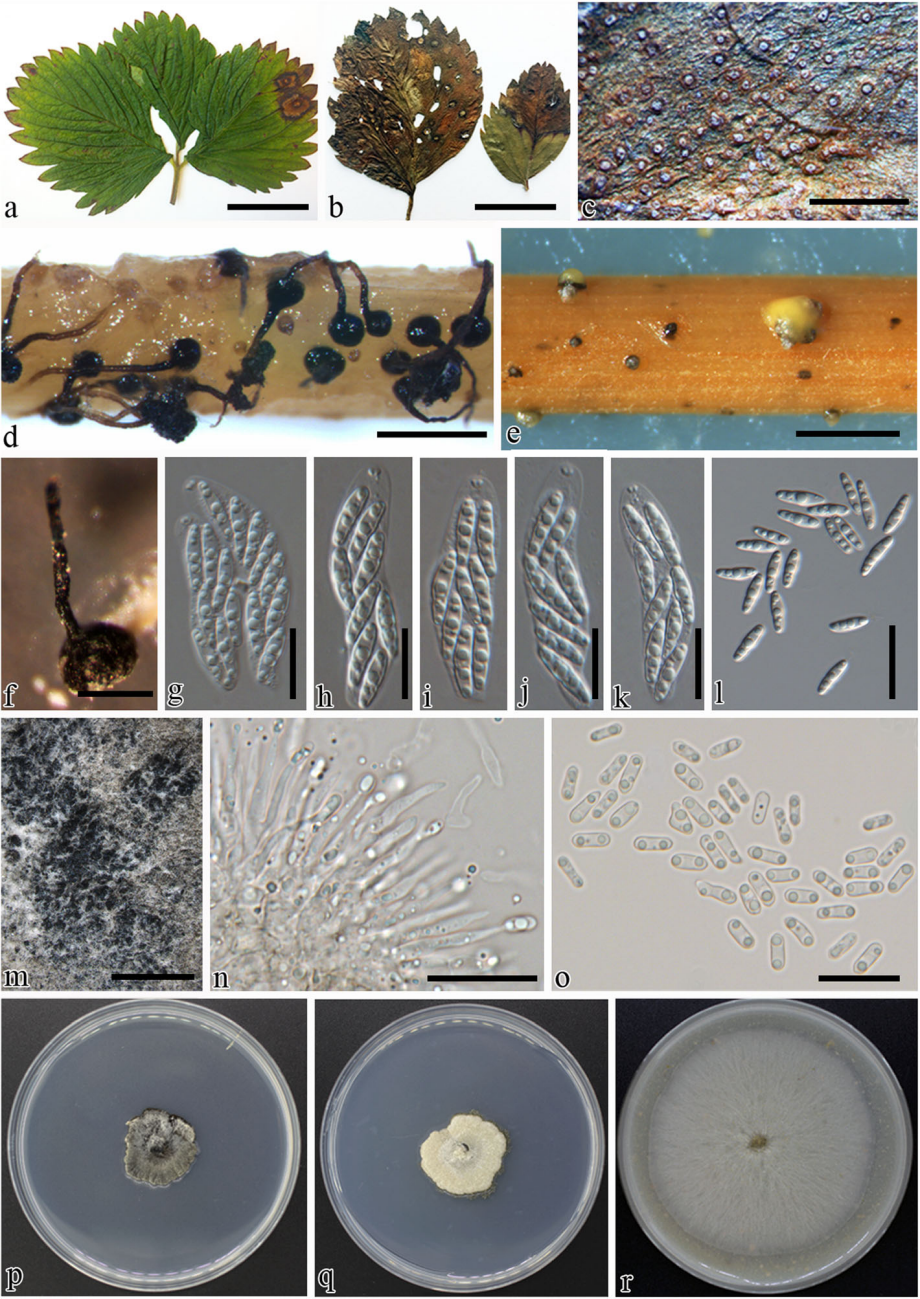
Morphology of *Gnomoniopsis fragariae* (BPI 877447, CBS 121226). **a,b** Infected leaves of *Fragaria* sp.. **c** Pycnidia on leaf surface. **d** Perithecia on alfalfa stems on WA. **e** Pycnidia on alfalfa stems on WA. **f** Single perithecium on WA. **g–k** Asci **l** Ascospores. **m** Pycnidia on culture. **n** Conidiophores. **o** Conidia. **p** 7-d-old culture on PDA. **q** 7-d-old culture on MEA. **r** 7-d-old culture on V8A. Scale bars: **a,b** = 3 cm, **c** = 300 μm, **d,e** = 800 μm, **f** = 200 μm, **g-l** = 10 μm, **m** = 600 μm, ***n*** = 15 μm, **o** = 12 μm

***Gnomoniopsis fragariae*** (Laib.) Udayanga & Castl. **comb. nov**. Fig. 3.

MycoBank: MB 835531.

*Basionym: Zythia fragariae* Laib., Arb. K. biol. Anst. f. Land-u-Forstwirt 6: 79–80. 1908.

*= Gnomonia fragariae* f. *fructicola* G. Arnaud, Traite de Pathologie Vegetale Encyclopedie Mycologique (Paris): 1558. 1931.

≡ *Gnomonia fructicola* (G. Arnaud) Fall, Can. J. Bot. 29: 309. 1951.

≡ *Gnomoniopsis fructicola* (G. Arnaud) Sogonov, Stud. Mycol. 62: 47. 2008.

= *Gloeosporium fragariae* G. Arnaud, Traite de Pathologie Vegetale Encyclopedie Mycologique (Paris): 1558. 1931.

= *Phyllosticta grandimaculans* Bubák & Krieg., in Bubák, Annls mycol. 10(1): 46. 1912.

*Type*: Illustration Abb. 3, page 80 (as *Zythia fragariae)* in Laibach (1908) Arbeiten aus der Kaiserlichen Biologischen Anstalt für Land- und Forstwirtschaft 6: 80 (**Lectotype** designated here; MBT 393837), Digitized by Universitätsbibliothek Johann Christian Senckenberg (UB Frankfurt am Main) and accessed here on 16 September 2020: http://www.digizeitschriften.de/dms/resolveppn/?PID=urn:nbn:de:hebis:30:4-16524, Image 114: Page 80). USA. MARYLAND: Beltsville, May 2006, *Turechek* (**Epitype** designated here BPI 877447, MBT 393837; ex-epitype culture AR 4275 = CBS 121226). GenBank: *ITS* = EU254824, *LSU* = EU255115, *TEF1* = EU221961, *RPB2* = EU219250.

*Description: Perithecia* on alfalfa stems black, solitary, superficial on substrate, globose, 200–250 μm diam, with long tapering neck co-occurring on stems and on WA with pycnidia together, multiple tapering perithecial necks protruding through substrata, 400–500 × 20–25 μm. *Asci* 29–33 × 6–9 μm (avg. ± SD = 31 ± 2 × 7 ± 1.5, *n* = 30), unitunicate, 8-spored, arranged obliquely uniseriate, irregularly biseriate or irregularly multiseriate, sessile or freely arranged, elongate to clavate, with conspicuous refractive ring at the apex. *Ascospores* 7–10 × 1.9–2.6 μm (avg. ± SD = 8.7 ± 0.7 × 2. 3 ± 0.2), hyaline, fusiform, one septate or bicellular, constricted at septum, 4-guttulate, and one cell is slightly smaller than the other.

*Pycnidia* on alfalfa stems on WA, globose, black, ostiolate, solitary, 50–100 μm diam, embedded in tissue, erumpent at maturity, with a short or inconspicuous neck, often with a yellowish, conidial cirrus extruding from ostiole; walls parenchymatous, consisting of 2–3 layers of medium brown textura angularis. *Conidiophores* 8–17 × 1–2.5 (avg. ± SD = 12 ± 2.5 × 2 ± 0.4), hyaline, smooth, unbranched or rarely branched at the base, ampulliform, long, slender and wider at the base. *Conidiogenous cells* phialidic, cylindrical, terminal, slightly tapering towards apex, 7–9 μm diam. Paraphyses absent. *Alpha conidia* 5.8–6.5 × 1.9–2.5 (avg. ± SD = 6 ± 0.4 × 2.2 ± 0.2), abundant in culture and on alfalfa stems, aseptate, hyaline, smooth, ellipsoidal to ovoid, biguttulate, base subtruncate, *Beta conidia* unknown.

*Culture* on PDA under artificial light at 25 °C for 1 wk., growth rate: 2.5 ± 0.2 mm/day (*n* = 3) white with irregular margins, in center with aggregations of mouse grey (118) crust like aerial mycelia with age or readily sporulating with yellow conidial cirri on black perithecia, dark mouse grey (119) pigmentation developing in reverse.

*Additional specimens examined*: FRANCE: Yvelines (formerly Seine-et-Oise), Chevreuse, on *Fragaria* sp., (date unknown), culture deposited 1934, G. Arnaud (CBS 208.34). Type of *Phyllosticta grandimaculans*: GERMANY: Sachsen, Königstein, on leaves of *Fragaria* sp., 1906–1912; W. Krieger, Krieger, Fungi Saxon. Exs. nr. 2179, (Krypto-S, F48606 **Lectotype** for *P. grandimaculans* designated here), ibid. (isotypes CUP, BPI 352482); DENMARK: Rindsholm, on leaves of *Fragaria* sp., 11 Oct. 1904, Lind J (BPI 352477).

*Geographic distribution*: Australia (Gomez et al. 2017), Belgium (Sogonov et al. 2008; Walker et al. 2010), Canada: British Columbia (Sogonov et al. 2008), China (Tai 1979); Denmark (this study), France (Sogonov et al. 2008; Walker et al. 2010), Germany (this study) Switzerland (Walker et al. 2010), Taiwan (Anonymous 1979), USA: Maryland, New York, Michigan (Alexopoulos and Cation 1952; Sogonov et al. 2008; Walker et al. 2010; Farr and Rossman 2020).

*Notes*: The name of the leaf blotch fungus was documented in phytopathological literature as *Gnomonia comari* (syn. *Gnomoniopsis comari*) before Sogonov et al. (2008) identified it as *Gnomoniopsis fructicola*. However, the earlier name *Zythia fragariae* (1908) represents the oldest name for this taxon as the asexual state of *G. fructicola* (Fall 1951). Although Arnaud (1931) identified the asexual state as a *Gloeosporium* sp., Fall (1951) mentions it as identical to *Z. fragariae*. Attempts to find type material for *Zythia fragariae* in European herbaria were unsuccessful. Therefore, the illustration available from the protologue is designated as a lectotype herein with a modern epitype designated. Microscopic observation of the isotype specimens of *Phyllosticta grandimaculans* housed in BPI, S and CUP and comparison of symptoms revealed that this species is conspecific with *Gnomoniopsis fragariae*. This pathogen appears to occur both in Europe and North America and is commonly associated with cultivated and wild species and varieties of *Fragaria* (Bolton 1954; van Adrichem and Bosher 1958; Maas 1998).

**Fig. 4:**
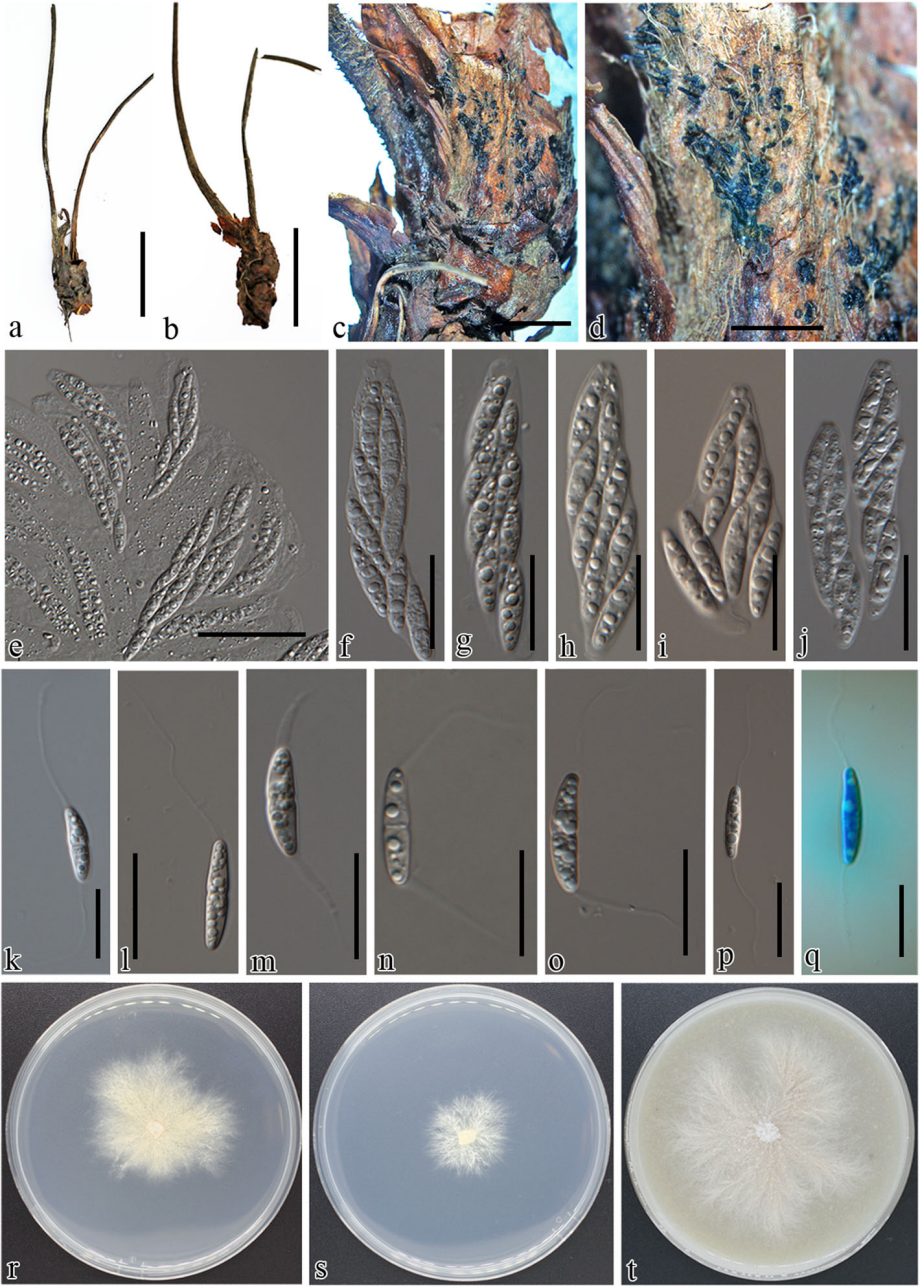
Morphology of *Paragnomonia fragariae* AG16076 (BPI 919211 and living culture CBS 143831). **a-d** Infected petioles of *Fragaria* sp. **e-j** Asci **k-q** Ascospores. **r** 7-d-old cultures on PDA. **s** 7-d-old cultures on MEA. **t** 7-d-old cultures on V8A. Scale bars: **a, b** = 2 cm, **c,d** = 1000 μm, **e** = 30 μm, **f-j** = 16 μm, **k,p,q** = 18 μm, **l–o** = 20 μm

***Paragnomonia fragariae*** (Kleb.) Senan. & K.D. Hyde, Mycosphere 8: 199. 2017. Fig. 4.

*Basionym: Gnomonia fragariae* Kleb., Haupt- und Nebenfruchtformen der Askomyzeten: Eine Darstellung eigener und der in der Literatur niedergelegten Beobachtungen über die Zusammenhänge zwischen Schlauchfrüchten und Konidienfruchtformen. 1: 285. 1918.

*Type*: Illustration Abb. 205, page 286., in H. Klebahn, Haupt-und Nebenfruchtformen der Askomyzeten: Eine Darstellung eigener und der in der Literatur niedergelegten Beobachtungenüber die Zusammenhänge zwischen Schlauchfrüchten und Konidienfruchtformen. 1918 (Lectotype designated by Moročko-Bičevska et al. (2019); Latvia: Tukums, Pūre, on dead petioles of *Fragaria × ananassa,* Lat: 57.0323418, Lon: 22.9160658, 20 Oct 2013, I. *Moroĉko-Biĉevska & J. Fatehi F129* [Epitype F367871(S); Iso-epitype DAU100004631 (DAU); ex-epitype culture F129/P3/1 = MSCL1603. *ITS* = MK524430, *LSU* = MK524447, *TEF1* = MK524466].

*Description: Perithecia* on crown and petioles of *Fragaria*, non stromatal, black, globose, arranged in immersed clusters on the base of the crown or solitary on petioles of the infected plants, 200–300 μm diam, bearing tapering black perithecial necks protruding from infected tissue 130–150 × 20–25 μm. *Asci* 50–60 × 8–10 (avg. ± SD = 56 ± 4 × 9 ± 1) μm unitunicate, 8-spored, sessile on defined hymenium or freely arranged with aging, elongate to clavate with conspicuous refractive ring at the terminals. *Ascospores* 14–17 × 3.5–5 (avg. ± SD = 16 ± 1.3 × 4 ± 0.4) μm, hyaline, fusiform to ellipsoid, straight to slightly curved, one septate or bicellular, with a conspicuous septum, slightly constricted at the septum, often 4-guttulate, two mucilaginous appendages present at the either ends of the ascospores. *Asexual morph* not seen in culture.

*Culture* on PDA under artificial light at 25 °C for 1 wk., growth rate: 2.8 ± 0.2 mm/day (*n* = 3) white, with sparse aerial mycelium, with irregular margins, rhizoid form of growth and in center and at edges with grayish yellow (57) pigmentation with age, dull green (70) pigmentation developing in reverse.

*Geographic distribution:* Confirmed distribution in Germany: Hamburg (Klebahn 1918), Switzerland:Vaud, Les Barges, Valais, Tessin (Bolay 1971; Monod 1983), United Kingdom, Latvia (all across the country), Sweden:Uppsala, Vastra (Moročko 2006; Moročko 2006), Lithuania:Kaunas, Siauliai and Finland: Parainen (Moročko-Bičevska et al. 2019), France (in this study).

*Additional specimens examined:* FRANCE: Côte-d’Or, Fontaine-Française, Le Revers des Lochères, on *Fragaria* sp. (cultivated), 20 May 2016, *Alain Gardiennet AG16076* (BPI 919211), GF 300 = M1530 = CBS 143831; Véronnes, 14 rue Roulette, on *Fragaria vesca*, 10 June 2012, *Alain Gardiennet AG12071* (BPI 919213); Bourberain, 37 route de Chazeuil, on *Fragaria* × *ananassa*, 10 June 2012, *Alain Gardiennet AG12072* (BPI 919212), Côte-d’Or, Bretenière, la Garande, on *Fragaria* sp. (cultivated), 23 June 2018, *Alain Gardiennet AG18036*, UK: on *Fragaria* sp. dates and *collector unknown* (IMI 10064, living culture CBS 146.64 = ATCC 16651).

*Notes:* The holotype specimen of *Gnomonia fragariae* was not available in Klebahn’s collection in BREM (pers. comm. Michael Stiller). The specimen (IMI 100647) linked to CBS 146.64 housed in K consists of a dry culture and slides, which may not contain conspicuous fungal structures to observe (pers. comm. Angela Bond & Paul Cannon); however, molecular data are available. Senanayake et al. (2017b) described the taxon without typifications and clarifications of affiliated names or specimens. Therefore, Moročko-Bičevska et al. (2019) designated original drawings by Klebahn (1918) specified in his original publication as a lectotype of *G. fragariae* and a freshly collected specimen from Latvia as an epitype, based on its morphology on the host and in culture.

## DISCUSSION

Post- and pre-harvest fungal diseases of strawberry cause significant annual losses to strawberry production (Maas 1998; Garrido et al. 2011). Phytopathogenic fungi are able to infect each and every part of the strawberry plant including leaves, petioles, fruits, sepals, stolon, crown and root systems at any age of the growth (Garrido et al. 2016). Although the fungal genera *Botrytis*, *Colletotrichum*, *Fusarium* and *Verticillium* causes major diseases of strawberry, several other pathogens also have significant impact on annual production (Leroch et al. 2013; Baroncelli et al. 2015). Species in the order *Diaporthales* also have been generally associated with strawberry diseases, although much confusion exists regarding the taxonomy, nomenclature, and evolutionary relationships of the taxa (Maas 1998; Garrido et al. 2016). In this study, evolutionary relationships of the leaf blight and leaf blotch pathogens widely known from North American strawberry fields and other strawberry growing regions in the world were revisited. Fresh collections of diseased specimens, pure cultures and multilocus phylogenetic analysis were used to resolve taxonomic problems. Type and other historic specimens from herbaria were observed and compared with fresh fungal collections to provide comprehensive nomenclatural clarification.

Leaf blight of strawberry was initially identified by characteristic large V-shaped necrotic lesions along major veins bearing black protruding necks of the pycnidia when examined under the stereo microscope (Fig. 2). Although the fungus infects leaves early in the growing season, leaf blight symptoms are more common on older leaves near or during harvest. The pathogen can weaken the plants through the destruction of older foliage and can also infect runner stems, calyxes, and fruits in some varieties (Maas 1998). The leaf blotch fungus, *Gnomoniopsis fragariae* is characterized by purplish to brown blotches and in later stages by large necrotic spots with abundant conidiomata around the major veins of the leaf (Fig. 3). The spots often occur on the end of a leaflet and are rounded to wedge shaped. This fungus can be found on the petiole, calyx, fruit stalk, and fruit. New collections of petiole blight and root rot pathogens were found in France occurring on stalks of perennial *Fragaria* sp. The symptoms often are confused with the early stages of leaf scorch caused by *Diplocarpon fragariae* (*Helotiales*) and leaf blotch caused by *G. fragariae*. Weakened plants may overwinter, which can result in reduced yields in the following season in commercial cultivations. Under conditions highly favorable for disease development, leaf blight can cause severe defoliation leading to plant death. Leaf blight fungus is often listed as a leading threat to strawberry and commonly co-occurs with other pathogens causing leaf blotch, leaf scorch and numerous leaf spots (Maas 1998). Close inspection of symptoms of various fresh specimens and historical collections housed in the U. S. National Fungus Collections revealed that it is possible to distinguish these taxa based on symptomology as well as microscopic examination of the fungal structures when present.

One additional species associated with strawberry included in the analysis is *Ophiogonomia rosae* (*Gnomoniaceae*) as identified by Walker et al. (2012) and represented by isolate CBS 128442 isolated from *Fragaria vesca* (Fig. 1). The same study reported the occurrence of *O. rosae* on overwintered leaves of *F. vesca*, *Comarum palustre*, *Rosa* sp., and *Rubus* sp. (*Rosaceae*) from various geographic regions of the world. Pathogenicity on these hosts is unknown, but it is likely *O. rosae* either possesses a saprobic lifestyle or is perhaps a weakly opportunistic pathogen. No specific reports of it as a pathogen of strawberry are known to exist and symptomology remains unknown.

The family composition of the order *Diaporthales* has changed with various classification systems originally based on morphology and later based on phylogenetic analyses (Wehmeyer 1975; Barr 1978; Castlebury et al. 2002), with *Diaporthaceae, Gnomoniaceae, Valsaceae, Melanconidaceae* and *Pseudovalsaceae* as the earliest defined families based on morphological characters (Wehmeyer 1975; Barr 1978; Vasilyeva 1987; Castlebury et al. 2002; Gryzenhout et al. 2006; Cheewangkoon et al. 2010; Crous et al. 2015). We confirmed that the *Melanconiellaceae*, which is broadly defined in this study, is a well-resolved family distinct from other closely related families. However, it is widely known that *Melanconium*-like taxa are polyphyletic and scattered throughout the order, and therefore need to be redefined with reference to the placement of the type species. The genus *Melanconiella* was considered as *Diaporthales incertae sedis* until recently and placed in *Sphaeriales* in early classifications (Clements and Shear 1931). It is now classified within *Melanconiellaceae* with numerous other species (Voglmayr et al. 2012; Du et al. 2017). *Melanconiella* species were known to be associated with the host family *Betulaceae*, including *Betula*, *Carpinus*, *Corylus* and *Ostrya*, and considered to be highly host specific. Du et al. (2017) described *M. cornuta* associated with canker and dieback of *Cornus controversa* (*Cornaceae*) and *Juglans regia* (*Juglandaceae*) from China. *Greeneria uvicola* causes bitter rot and necrotic fleck of grapes (*Vitis* spp., *Vitaceae*) in North America, Australia and elsewhere in the world and often misidentified and is often confused with other common diaporthalean pathogens on grapevines including *Diaporthe ampelina* (*Diaporthaceae*) (Farr et al. 2001; Steel et al. 2007; Longland and Sutton 2008). *Microascospora rubi* is associated with *Rubus ulmifolia* from Italy but appears to be a saprobe (Senanayake et al. 2017a). However, the generic delimitation and species diversity within the family *Melanconiellaceae* are yet to be resolved with more collections and molecular data of closely related taxa.

Early morphology-based classification systems placed species that occur singly within the substrate without any stromatic development in the family *Gnomoniaceae* (Wehmeyer 1975; Barr 1978; Monod 1983). However, molecular data and large-scale sampling of taxa have revealed that gnomoniaceous taxa sensu Wehmeyer (1975) and Monod (1983) are polyphyletic. Improvements in phylogenetic understanding have ultimately resulted in a more natural classification, leading to better insights into the evolutionary history of the Diaporthales and other Sordariomycetes (Zhang et al. 2006; Hongsanan et al. 2017; Guterres et al. 2019). These methods have also led to improvements of the understanding of the seemingly minor morphological differences of the sexual morphs of these ascomycete genera for identification purposes. Therefore, finding and utilizing phylogenetically informative genes are critical to obtain compelling, yet previously unrecognized, data to develop new evolutionarily significant insights and to encourage innovative practices in modern fungal systematics.

Due to the morphological plasticity of both asexual and sexual morphs, confusion has remained in generic and family-level classifications of many diaporthalean fungi. Phylogenetic analyses based on single gene trees have been often problematic. The conventionally used nuc 28S rDNA roughly distinguished taxa at generic and family levels, but several genera and families were poorly supported or otherwise not distinguished. Single morphological characters previously used to segregate genera or families in ascomycetes have often been found to be discordant with multilocus phylogenies and phylogenomic analyses (Choi and Kim 2017; Yang et al. 2018; Voglmayr et al. 2019b).

The best approach for developing knowledge about species in this diverse group of plant-associated fungi is through a consolidated platform utilizing morphological data, multigene phylogeny, as well as host associations and historical background information connected to voucher specimens in herbaria. For instance, correct identification of *Paraphomopsis obscurans* required the time-consuming process of sifting through the complicated historical literature of various genera within *Diaporthales* as well as unrelated genera and observation of numerous specimens. From this historical research, it was evident that previous authors observed morphological and physiological distinctions from other genera including *Dendrophoma*, *Diaporthe*, *Phoma*, *Phyllostica*, *Sphaeronaemella*, and *Zythia.* As the taxonomic opinions were based on the observation of the vouchered specimens, it was possible to reassess these opinions based on the same or other authentic specimens. To this end, a consolidated approach of multilocus phylogenetic analyses and morphological observations will provide the best resolution for taxonomists, evolutionary biologists, plant pathologists, and quarantine officials in their efforts to address issues regarding accurate identification, host plant associations and interactions, and disease management.

## CONCLUSIONS

Molecular phylogeny based on newly generated DNA sequences of diaporthalean fungi associated with strawberry diseases revealed that the leaf blight pathogen represents a new evolutionary lineage within the family *Melanconiellaceae*, distinct from closely related taxa. The combined phylogeny based on four loci (*ITS*, *LSU*, *RPB2*, and *TEF1*) together with morphological data illustrate the generic and family-level relationships in this diverse order of fungi. Although, leaf blight, leaf blotch, petiole blight and root rot fungi of strawberry are frequently encountered, the taxonomy, accurate naming and geographic distribution were largely overlooked until recently. Therefore, this study highlights the need for revisiting poorly known genera of phytopathogenic diaporthalean fungi in order to establish their evolutionary relationships and provide reference DNA sequences for accurate identification purposes.

## Data Availability

The datasets generated and analysed during the current study are available in the Ag Data Commons, U.S. Department of Agriculture 10.15482/USDA.ADC/1518737

## ABBREVIATIONS

avg.: Average
BI: Bayesian Inference
BPI: United States National Fungus Collections
BPP: Bayesian Posterior Prabalities
ITS: Ribosomal internal transcribed spacers 1 and 2 with 5.8S ribosomal DNA
LSU: 28S ribosomal DNA/large subunit rDNA
MEA: Malt Extract Agar
ML: Maximum Likelihood
nuc 18S rDNA: Nuclear 18S/ small subunit of ribosomal DNA
PDA: Potato Dextrose Agar
rDNA: Ribosomal DNA
5.8S: Ribosomal DNA 5.8S region
RPB2: Partial sequences of second largest subunit of RNA polymerase II
SD: Standard Deviation
TEF1: Translation elongation factor 1-α
V8A: V8 juice Agar
WA: Water Agar
wk: Week
